# LC-MS/MS Quantification of Nevirapine and Its Metabolites in Hair for Assessing Long-Term Adherence

**DOI:** 10.3390/molecules25235692

**Published:** 2020-12-02

**Authors:** Haoran Yang, Liuxi Chu, Yan Wu, Wei Wang, Jin Yang, Quan Zhang, Shan Qiao, Xiaoming Li, Zhiyong Shen, Yuejiao Zhou, Shuaifeng Liu, Huihua Deng

**Affiliations:** 1 Key Laboratory of Child Development and Learning Science, Ministry of Education, Southeast University, Nanjing 210096, China; 220174280@seu.edu.cn (H.Y.); clx@seu.edu.cn (L.C.); rclswy@seu.edu.cn (Y.W.); 220181839@seu.edu.cn (W.W.); yangjin@seu.edu.cn (J.Y.); 2 Department of Brain and Learning Science, School of Biological Sciences & Medical Engineering, Southeast University, Nanjing 210096, China; 3 Institute of Child Development and Education, Research Center of Learning Science, Southeast University, Nanjing 210096, China; 4 School of Public Health, Southeast University, Nanjing 210009, China; 5 Department of Health Promotion, Education and Behavior, South Carolina Smart State Center for Healthcare Quality (CHQ), University of South Carolina, Columbia, SC 29208, USA; zhangquan02@163.com (Q.Z.); shanqiao@mailbox.sc.edu (S.Q.); xiaoming@mailbox.sc.edu (X.L.); 6 Institute of Applied Psychology and School of Public Administration, Hohai University, Nanjing 211100, China; 7 Guangxi Center for Disease Control and Prevention, Nanning 530028, China; shenzhiyong99999@sina.com (Z.S.); gxcdcj2013@163.com (Y.Z.); liushuaifeng@163.com (S.L.)

**Keywords:** nevirapine, 2-hydroxynevirapine, 3-hydroxynevirapine, LC-MS/MS, hair, long-term adherence

## Abstract

The adherence assessment based on the combination of nevirapine (NVP) and its two metabolites (2-hydroxynevirapine and 3-hydroxynevirapine) would more comprehensively and accurately reflect long-term adherence than that of a single prototype. This study aimed to develop a specific, sensitive and selective method for simultaneous detection of the three compounds in hair and explore whether there was consistency among the three compounds in assessing long-term adherence. Furthermore, 75 HIV-positive patients who were taking the NVP drug were randomly recruited and divided into two groups (high-and low-adherence group). All participants self-reported their days of oral drug administration per month and provided their hair strands closest to the scalp at the region of posterior vertex. The concentrations of three compounds in the hair were determined using a developed LC-MS/MS method in multiple reaction monitoring. This method showed good performances in limit of quantification and accuracy with the recoveries from 85 to 115% and in precision with the intra-day and inter-day coefficients of variation within 15% for the three compounds. The population analysis revealed that patients with high-adherence showed significantly higher concentrations than those with low-adherence for all three compounds. There were significantly moderate correlations of nevirapine with 2-hydroxynevirapine and 3-hydroxynevirapin and high correlation between 2-hydroxynevirapine and 3-hydroxynevirapin. The two NVP’s metabolites showed high consistency with NVP in evaluating long-term adherence.

## 1. Introduction

Non-nucleoside reverse transcriptase inhibitors (NNRTIs) have turned into a necessary component of the drug combination schemes that are presently utilized in the therapy of human immunodeficiency virus (HIV) infections [1,2,3]. NNRTIs can bind reversibly to specific points on human immunodeficiency virus reverse transcriptase and can terminate RNA- and DNA-dependent DNA polymerase activity, thereby preventing virus replication. Nevirapine (NVP), the first-generation NNRTI, in alliance with other antiretroviral drugs has been widely used to decrease the morbidity and improve health outcomes of people living with HIV [4,5,6,7].

On the other hand, the ultimate therapeutic effect in the prevention and treatment of acquired immune deficiency syndrome not only depends on the drug regimen, but also requires HIV-infected people to ensure strict long-term adherence to antiretroviral drugs [8]. Low adherence is generally considered to result in adverse clinical outcomes [9]. Evaluation of patients’ adherence to antiretroviral drugs is helpful in predicting virology failure, avoiding drug resistance, grasping the degree of disease deterioration and ensuring the effect of prevention and treatment [10].

Previous studies mostly utilized drug concentrations in plasma and urine as biomarkers to assess the adherence to antiretroviral drugs [11,12]. Although these biomarkers may be sensitive, both of them can reliably provide only the adherence within several hours or a few days, not for the adherence over a relatively long period, such as one month or half a year. The drug concentration in the 1-cm hair nearest the scalp can be traced back to drug use within the past month if the hair grows 1 cm each month. Thus the concentrations of drugs in hair matrix resulting from passive diffusion of plasma species could be adopted as evaluation of long-term adherence to antiretroviral drugs [13,14]. Earlier studies have demonstrated that the concentration of antiretroviral drugs in hair is a reliable measure for assessment of long-term adherence to antiretroviral drugs [15,16,17,18,19,20,21].

Existing studies utilize the concentrations of the drugs’ prototype in hair to evaluate long-term adherence [22,23,24,25]. However, the antiretroviral drugs are mostly metabolized in the circulating system once orally administered and only a fraction of them remain as the prototype structure. For example, NVP is extensively transformed under the catalysis of internal cytochromes (e.g., CYP3A, CYP2B6, CYP2D6 and CYP3A4) into some hydroxylated metabolites including 2-hydroxynevirapine (2-OH NVP), 3-hydroxynevirapine (3-OH NVP), 8-hydroxynevirapine (8-OH NVP) and 12-hydroxynevirapine (12-OH NVP). Among these metabolites, 2-OH NVP is exclusively formed by CYP3A and 3-OH NVP is exclusively formed by CYP2B6 [26]. The formations of 8-OH NVP and 12-OH NVP are attributed to the catalysis of both CYP2D6 and CYP3A4 [27]. Indeed, most of NVP is metabolized into metabolites after being exposed to the body, among which 2-OH NVP and 3-OH NVP are the main metabolites. This was demonstrated by the previous findings that no more than 5% of NVP was excreted unaltered in the urine after the metabolism in the body, but 2-OH NVP and 3-OH NVP were excreted at 22.9 and 33.1% as detected by a radio-labeled mass balance [28,29]. Thus, the content of the drug prototype in biological matrix (e.g., plasma and hair) cannot fully reflect the concentration information about the drug which HIV patients took by oral administration. Additionally, there is large inter-individual difference in drug metabolism. For example, coefficients of variation (CV) in the metabolism of NVP, lamivudine and tenofovir were 99.1, 75.4 and 103.8% as shown by the previous study [30]. Therefore, the adherence assessment based on the drug prototype in hair might be less accurate. Alternatively, the assessment based on the combination of NVP and its main metabolites would more comprehensively and accurately reflect long-term adherence. Consequently, it is required to develop a sensitive and selective method for simultaneously determining NVP and its two main metabolites, 2-OH NVP and 3-OH NVP in hair.

LC-MS/MS has been widely applied because it has high sensitivity and selectivity [12,30]. Previous studies have developed several LC-MS/MS methods for the determination of NVP alone or together with some other antiretroviral drugs in hair [31,32,33]. Some rapid and highly sensitive LC-MS/MS methods have also been established for quantifying NVP and its metabolites in human serum and baboon plasma [28,34,35]. However, there was no study to report determination of NVP’s main metabolites, 2-OH NVP and 3-OH NVP, in hair and simultaneous determination of NVP and the two main metabolites. On the other hand, atmospheric pressure chemical ionization (APCI) was generally suitable for the detection of low-polar and non-polar compounds and electrospray ionization (ESI) was applied to detect a wider range of compounds from low-polar to polar compounds although they are the ionization techniques most commonly used for the LC-MS/MS quantification. However, most of the previous studies selected ESI as an ion source to detect NVP and its metabolites together in plasma that were of low polarity. For example, Ren et al. developed a sensitive LC-MS/MS method with ESI for quantification of nevirapine and its metabolites in baboons [34]. Liu et al. also developed an ESI^+^-based LC-MS/MS method for quantitation of nevirapine and its two oxidative metabolites in baboons [35]. In fact, the ESI-based method has some limitations compared to the APCI-based method. In detecting low polarity compounds, ESI often caused higher background noise than APCI because ESI is more susceptible to matrix effects [36]. Additionally, raw samples need to be preprocessed with complicated steps to reduce high background noise when ESI is used. This undoubtedly added to the difficulty of handling the sample. For example, Huang et al. developed a sensitive LC-MS/MS method with ESI for quantification of nevirapine in human hair. In addition to the routine incubation process with mobile phase, the liquid-liquid extraction step was also added to reduce background noise in their process of handling the hair sample. The specific operation is that NVP in hair was extracted by liquid-liquid extraction with sodium phosphate buffer, methyl *tert*-butyl ether (MTBE) and ethyl acetate (EA) [33]. Obviously, a complicated sample pretreatment process (e.g., the liquid-liquid extraction) brings unnecessary environmental pollution and disposal cost due to the solvent evaporation. By contrast, an LC-MS/MS method based on APCI would not need redundant handling processes because the use of APCI often caused relatively lower background noise than ESI, following lower matrix effect [37]. So APCI would be a better choice for simultaneous analysis of NVP, 2-OH NVP and 3-OH NVP in hair.

The present study firstly aimed to develop the LC-APCI-MS/MS method with high specificity, sensitivity and selectivity for simultaneous detection of NVP, 2-OH NVP and 3-OH NVP in hair. Then the developed method would be put into exploring the consistence in assessing long-term adherence of NVP through examining associations between patients’ self-reported adherence and the concentrations of the three compounds in hair.

## 2. Results

### 2.1. Chromatography

**Figure 1:**
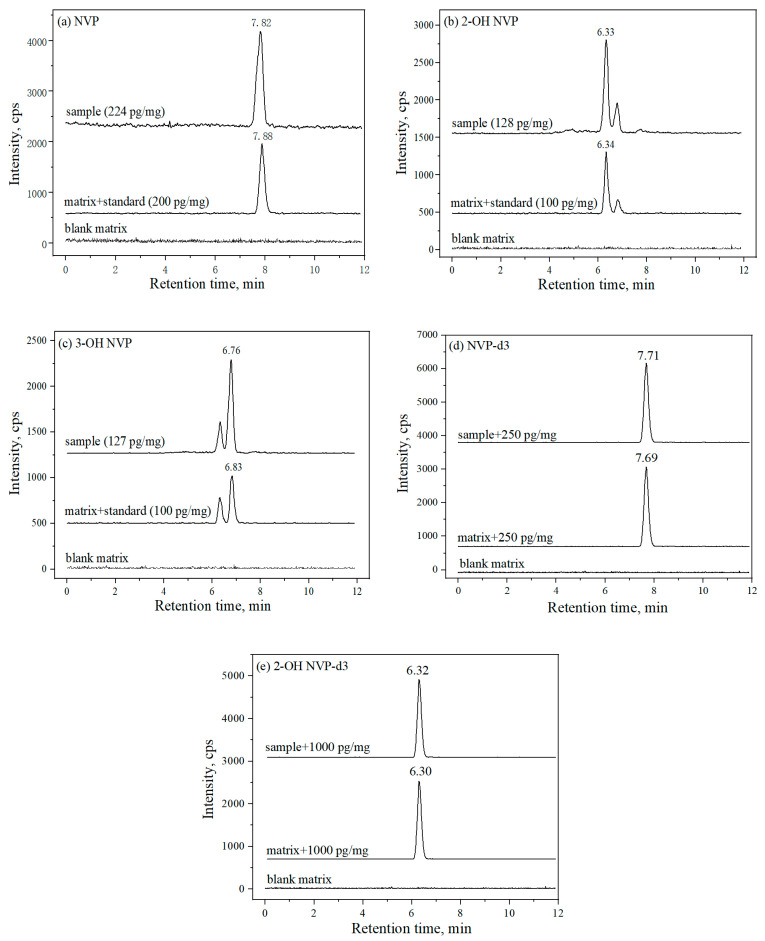
The chromatograms of the three analysts, (**a**) nevirapine (NVP), (**b**) 2-OH NVP and (**c**) 3-OH NVP in blank hair matrix, blank hair matrix spiked into standard solutions and natural hair samples from HIV patients who took NVP. As spiked into hair blank matrix, concentration of standards were 200 pg/mg for NVP, 100 pg/mg for 2-OH NVP and 3-OH NVP. The representative concentrations of drugs determined in natural hair samples were 224 pg/mg for NVP, 128 pg/mg for 2-OH NVP and 127 pg/mg for 3-OH NVP. The internal standard’s (IS’s) concentrations were 250 pg/mg for (**d**) NVP-d3 and 1000 pg/mg for (**e**) 2-OH NVP-d3, which spiked into blank hair matrix and natural hair samples.

The NVP, 2-OH NVP and 3-OH NVP and their corresponding internal standard (IS) were separated and well resolved by observing their chromatographic peaks in Figure 1. Under the chromatographic condition; NVP, 2-OH NVP and 3-OH NVP were eluted at 7.84, 6.41 and 6.92 min and their ISs, NVP-d3 and 2-OH NVP-d3 were eluted at 7.69 and 6.30 min, respectively. In addition, all analytes in patients’ hair samples were also well resolved in the same way.

### 2.2. Method Validation

The method had good linearity within the set concentration range showing that the square of correlation coefficient (*R*^2^) of the calibration curve was more than 0.99 for all three compounds in hair. Limits of detection (*LOD*s) were 3, 2 and 2 pg/mg for NVP, 2-OH NVP and 3-OH NVP. Limits of quantitation (*LOQ*s) were 11, 6 and 6 pg/mg for NVP, 2-OH NVP and 3-OH NVP. Intra-day and inter-day CVs were less than 15% for the three compounds, and the recovery was in the range of 85–115% as listed in Table 1. From these parameters, which were consistent with the guidelines, it turned out that the method had good precision and accuracy for analyzing the three compounds in hair.

The means of matrix factor (*MF)* and IS normalized *MF* were close to 1 for NVP, 2-OH NVP and 3-OH NVP, and the CVs of *MF* and IS normalized *MF* were less than 15% for all three compounds (the details seen in Table S1 in Supplementary Materials). The CVs of the selectivity for external standards (ESs) were within 20% and the CVs of the selectivity for IS were within 5% (the details seen in Table S2 in Supplementary Materials), indicating that all analytes showed good selectivity. The stability assessments of NVP, 2-OH NVP and 3-OH NVP revealed that the deviation against the nominal concentrations and CVs at each level were less than 15% in different anticipated conditions (details seen in Table S3).

### 2.3. Detection of HIV Patients’ Hair Samples

The chosen biological matrix was the 1-cm hair segment closest to the scalp, which was collected from 75 HIV-positive patients who had self-reported taking NVP in the past month. As listed in Table 2, HIV patients with high adherence to antiretroviral drugs obviously showed more days of NVP oral administration than those with low adherence (*p* < 0.001) and included less females (*p* < 0.01), but there was no difference between the two groups in age (*p* > 0.05). They displayed significantly higher concentrations than those with low adherence for all three compounds in hair (*p*s < 0.001). Univariate analysis of variance further revealed that the differences remained significant for all three compounds in hair (*F*_1,72_ = 45.719, *η*_p_^2^ = 0.388, *sp* = 1.000, *p* < 0.001 for NVP, and *F*_1,72_ = 13.791, *η*_p_^2^ = 0.161, *sp* = 0.956, *p* < 0.001 for 2-OH NVP, and *F*_1,72_ = 15.639, *η*_p_^2^ = 0.178, *sp* = 0.974, *p* < 0.001 for 3-OH NVP) after gender was used as a covariate. These results indicated that there were high consistencies in association with patients’ self-reported adherence among the three compounds in hair. Additionally, gender had no influences on NVP and 2-OH NVP (*p*s > 0.107) and might influence 3-OH NVP (*F*_1,72_ = 4.083, *η*_p_^2^ = 0.054, *sp* = 0.513, *p* = 0.047). Furthermore, there were no gender differences in the high adherence group in age (*Z* = −0.116, *p* = 0.907), days of NVP oral administration (*Z* = −0.634, *p* = 0.526) and the concentrations of NVP and its metabolites (*Z* = −0.294, *p* = 0.769 for NVP, and *Z* = −1.109, *p* = 0.268 for 2-OH NVP and *Z* = −1.584, *p* = 0.113 for 3-OH NVP). In the low adherence group, there were also no gender differences in age (*Z* = −1.688, *p* = 0.091), days of NVP oral administration (*Z* = −0.796, *p* = 0.426) and the concentrations of the three compounds (*Z* = −0.129, *p* = 0.897 for NVP, and *Z* = −1.428, *p* = 0.153 for 2-OH NVP and *Z* = −0.264, *p* = 0.792 for 3-OH NVP).

**Figure 2:**
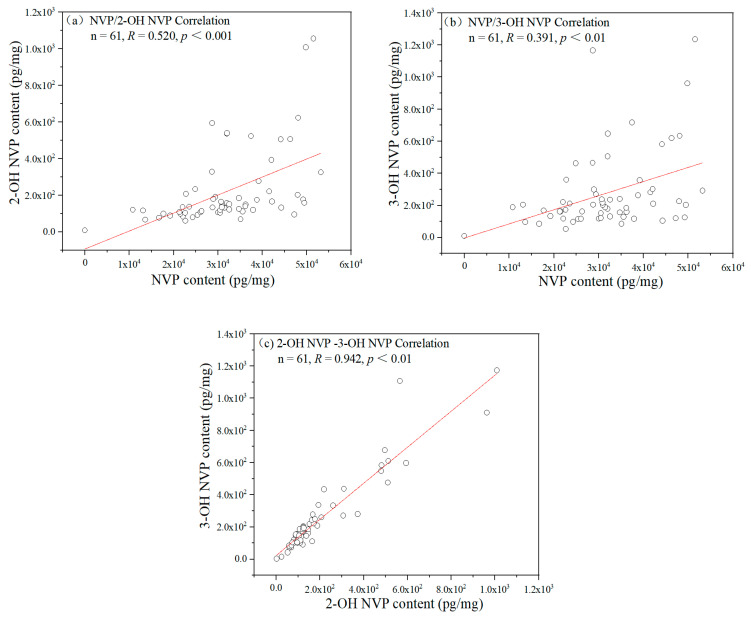
The correlations in hair concentration among NVP, 2-OH NVP and 3-OH NVP. (**a**) NVP and 2-OH NVP, (**b**) NVP and 3-OH NVP and (**c**) 2-OH NVP and 3-OH NVP among 61 patients with the concentrations of all three compounds in hair over *LOQ*. *R* is the coefficient of correlation and *p* represents statistical significance in the figure.

The NVP concentrations in hair showed significantly moderate correlations with the concentrations of 2-OH NVP and 3-OH NVP in hair among 75 patients (*R* = 0.617 and *R* = 0.557, *p*s < 0.001), but there was significantly high correlation between 2-OH NVP and 3-OH NVP (*R* = 0.951, *p* < 0.001). Even if 14 participants with hair concentrations of any one of the three compounds below *LOQ* were excluded, there remained moderate correlations of NVP with 2-OH NVP and 3-OH NVP (*R* = 0.520, *p* < 0.001 and *R* = 0.391, *p* < 0.01) and high correlation between 2-OH NVP and 3-OH NVP (*R* = 0.941, *p* < 0.001) among the remaining 61 patients as shown in Figure 2.

## 3. Discussion

We developed an LC-APCI^+^-MS/MS method with high specificity, sensitivity and selectivity for simultaneous quantification of NVP and its two metabolites, 2-OH NVP and 3-OH NVP, in hair. As far as we know, this was the first successful attempt to determine 2-OH NVP and 3-OH NVP and evaluate long-term adherence based on the concentrations of metabolites in human hair. Previous studies were limited to the detection of NVP metabolites in other biological samples, such as human serum [28] and for the adherence evaluation based on drug prototypes in hair [22,23,24,25]. Additionally, we found that NVP’s metabolites showed high consistency with NVP in evaluating long-term adherence. Specifically, patients with high adherence showed significantly higher concentrations of NVP, 2-OH NVP and 3-OH NVP in hair than those with low adherence. We discuss each of the results in turn below.

The present method showed *LOQ* at 11 pg/mg for NVP and 6 pg/mg for both 2-OH NVP and 3-OH NVP. In a previous study, the *LOQ* at 250 pg/mg for NVP using the LC-ESI-MS/MS method with a mobile phase was composed of 50% acetonitrile. In their study, routine incubation with methanol/trifluoroacetic acid (9:1, *v*/*v*) and liquid-liquid extraction was used during the hair sample processing prior to mass spectrometry analysis [33]. In another previous study, they used the LC-ESI-MS/MS method to quantitate NVP with a mobile phase composed of 80% methanol. The *LOQ* of NVP is 39 pg/mg. In their study, the routine incubation with methanol was used during the hair sample processing before mass spectrometry analysis [30]. The *LOQ* at 11 pg/mg for NVP in the present LC-APCI-MS/MS was better than that at 250 and 39 pg/mg in the previous LC-ESI-MS/MS.

This was consistent with the previous observation that matrix had less effect on APCI source than ESI [38,39,40], although the occurrence of matrix effects was observed in both ESI and APCI. In other words, compared with ESI, using APCI was less likely to produce higher background noise, thereby resulting in lower *LOQ*s. On the other hand, the assay method utilized a mixture of methanol with water (78:22, *v*/*v*) as mobile phase for the easily chromatographic separation of NVP and its metabolites (2-OH NVP and 3-OH NVP) that had higher polarity than NVP. Besides, *LOQ* at 6 pg/mg was attained for 2-OH NVP and 3-OH NVP, demonstrating that the present method had good sensitivity for the two metabolites in hair.

This study found that the NVP concentrations in the hair from patients with high adherence were significantly higher than those from patients with low adherence. Previously, the concentrations of drug prototypes, zidovudine and efavirenz, in hair were also found to be closely associated with long-term adherence to the antiretroviral therapy [31]. This study also found that the concentrations of 2-OH NVP and 3-OH NVP in the hair from patients with high adherence were significantly higher than those from patients with low adherence. These results indicated that NVP’s metabolites showed high consistency with NVP in evaluating long-term adherence. Therefore it inferred that the adherence evaluation based on the combination of the antiretroviral drug’s prototype with its metabolites in hair would be more comprehensive and accurate.

This study also found that there were significantly positive correlations of NVP with 2-OH NVP and 3-OH NVP and correlation between the two metabolites. However, the coefficient of correlation between the two metabolites was larger than that between NVP and the two metabolites (*R* = 0.941 vs. *R* = 0.520 and *R* = 0.391). This was mainly due to the large differences in the physiochemical properties between NVP and the two metabolites. As region-specific isomers, both 2-OH NVP and 3-OH NVP have one hydrophilic group, hydroxyl, thereby having the same or similar physiochemical properties (e.g., polarity, hydrophilicity and basicity). As a result, 2-OH NVP and 3-OH NVP maybe have the same incorporation pattern into hair and mechanism of dissolution from hair, resulting in significantly high correlation between them. Previously, high correlation was also observed for correlation between the hair concentrations of lopinavir and ritonavir which had similar physical and chemical properties [31]. Compared to the two metabolites, NVP lacked one hydrophilic group and was a highly lipophilic molecule [6]. Thus, NVP could show a different incorporation pattern into hair and mechanism of dissolution from the hair matrix, resulting in moderate correlations with the two metabolites. In addition, NVP has less polarity and higher lipophilicity than the two metabolites. The lipophilicity of the substance itself is one of key factors influencing the drug’s incorporation into hair [14]. Thus, NVP with stronger lipophilicity more easily penetrates cell membranes into the stromal cell depending on the concentration gradient. Therefore, this study found that the NVP concentration in hair is much higher than that of its two metabolites in hair. Thus, the concentrations of the metabolites in hair would be a valuable clinical reference for the NVP concentration in hair in evaluating patients’ long-term adherence.

## 4. Materials and Methods

### 4.1. Standard Substances and Solution Preparation

Standard, NVP was attained from TargetMol (Shanghai, China). Furthermore, 2-OH NVP and 3-OH NVP were supplied by Toronto Research Chemicals (Toronto, ON, Canada). NVP-d3 as internal standard (IS) of NVP and 2-OH NVP-d3 as IS of 2-OH NVP and 3-OH NVP were supplied by Toronto Research Chemicals (Toronto, ON, Canada). Ammonium acetate of LC-MS grade was obtained from Tedia (Fairfield, OH, USA), methanol of LC-MS grade was obtained from Sigma Aldrich (St. Louis, MO, USA). Deionized water was purchased from A.S. Watson Group (Hong Kong, China).

NVP, 2-OH NVP and 3-OH NVP were separately dissolved in methanol as stock solutions at concentrations of 1 mg/mL, 0.5 mg/mL and 50 μg/mL, respectively. The mixed working solution of the three compounds was needed to dilute the stock solution to the expected concentrations with methanol, such as 0.8–10,000 ng/mL for NVP and 0.4–250 ng/mL for 2-OH NVP and 3-OH NVP. The IS working solutions with high concentration were diluted with methanol to 50 ng/mL of NVP-d3 and 200 ng/mL of 2-OH NVP-d3. All of the above stock solutions were stored in the refrigerator.

### 4.2. Hair Collection

Seventy-five HIV-positive patients who were taking NVP were recruited from Guangxi Zhuang Autonomous Region in China. All of them signed written informed consent before the study. The study was permitted by the Institutional Review Board (IRB) at the Guangxi Center of Disease Control in China and the University of South Carolina, USA. This study was also authorized by the health science research ethics committee of Southeast University and followed the Declaration of Helsinki.

With the help of some local research volunteers, the patients completed a detailed questionnaire including their demographics and the number of days they had taken NVP orally in the past 30 days. Every patient in the study should take 200 mg of NVP twice a day following the doctor’s advice. All the participants were from Guangxi province. Besides, all participants provided their hair strands closest to the scalp at the region of posterior vertex. Hair samples were sealed with foil once collected, thereby avoiding direct irradiation by sunlight, and then were stored in a dry and dark environment at room temperature until the analysis. In order to match the time span of questionnaire measurements with information from the past month, we used only the 1-cm hair fragment nearest the scalp in the next LC-MS/MS analysis.

### 4.3. Hair Incubation

The 1 cm long hair strands were cleaned twice with methanol for 2 min and dried for 20 min under the condition of drying air, they were then cut into pieces (1–2 mm) with clean scissors. After being weighed at 10 mg, hair pieces were transferred to a clean test tube. Next, 950 μL methanol and 50 μL IS working solution were added to the test tube with hair samples. Subsequently, the mixture was wholly vortex-mixed for 60 s, and incubated for 16 h at 37 °C in an electric-heated thermostatic water bath. After 2 min in the vortex machine and 5 min at 12,000 rpm in the centrifuge, 800 μL supernatant was transferred to another clean test tube and evaporated with pure nitrogen gas at 50 °C. Finally, the residue was dissolved with 50 μL mobile phase for mass-spectrometric detection.

### 4.4. LC-MS/MS Analysis

Chromatographic separation of the three compounds and their ISs was implemented on an Agilent 1200 HPLC system (Waldbronn, Germany). The injection volume into an LC-MS/MS system was 20 μL. The LC system was equipped with the Platisil ODS C18 reverse phase analytical column (150 mm × 4.6 mm, 5 μm; Dikma, Beijing, China) which was protected by a C18 guard cartridge (10 mm × 4.6 mm, 5 μm; Dikma). In addition, the mobile phase was made of a mixture of methanol and distilled water (78:22, *v*/*v*) with ammonium acetate (1 mM). Then it was filtered with a micro-porous membrane to remove the tiny particles and treated with ultrasonic process for 20 min to eliminate air bubbles. The flow rate was set at 500 μL/min.

**Figure 3:**
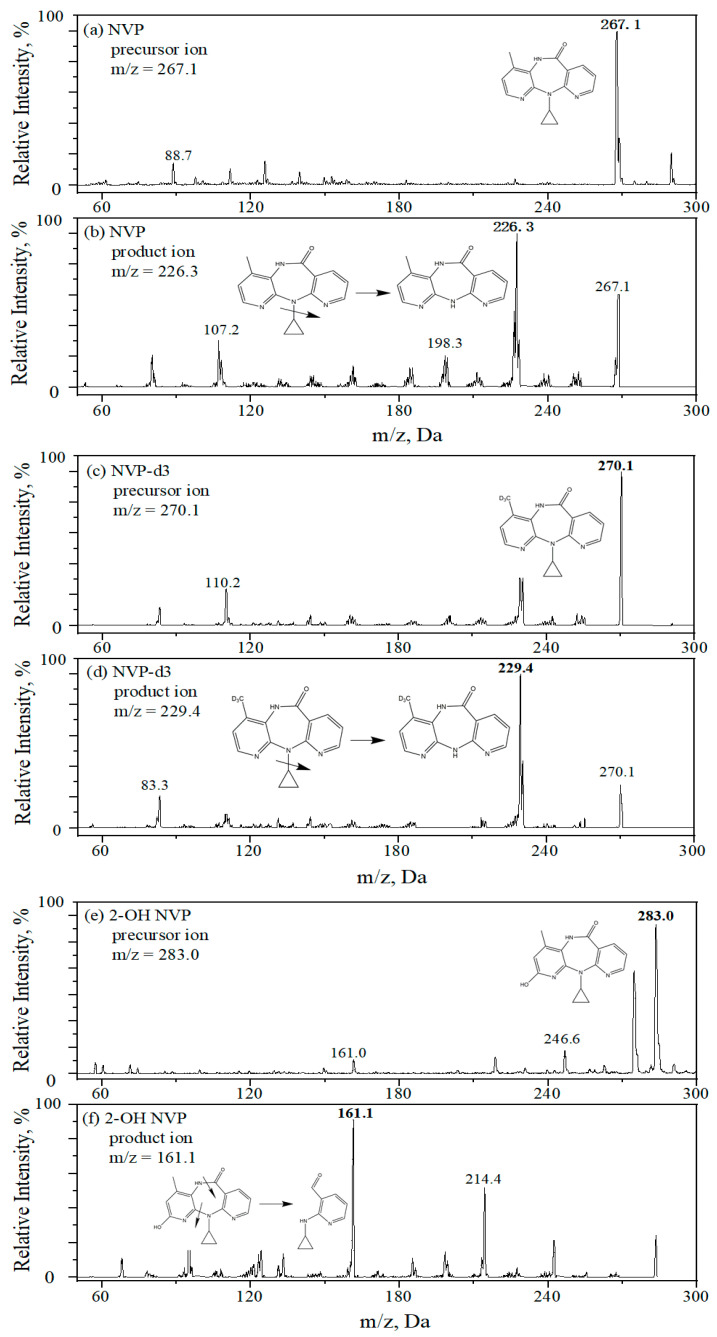

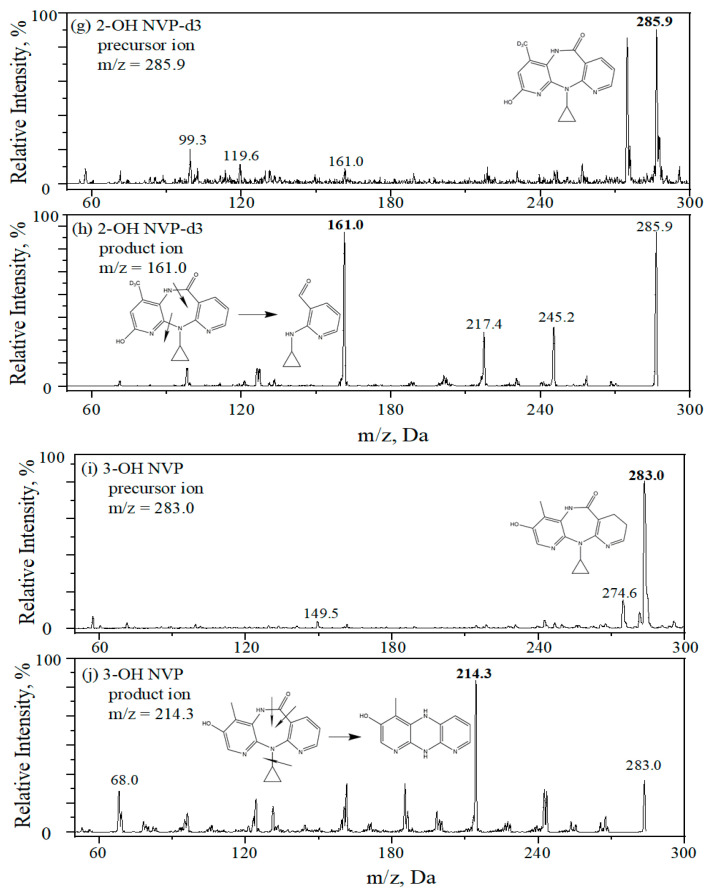
Precursor ion (**a**,**c**,**e**,**g**,**i**) and product ion (**b**,**d**,**f**,**h**,**j**) of analytes and internal standards, (**a**,**b**) NVP, (**c**,**d**) NVP-d3, (**e**,**f**) 2-OH NVP, (**g**,**h**) 2-OH NVP-d3 and (**i**,**j**) 3-OH NVP.

MS/MS analysis was performed on a QTRAP 3200 mass spectrometer (AB Sciex, Foster City, CA, USA) using an APCI with positive mode and in multiple reaction monitoring (MRM). Nitrogen (99.99%) was used as a nebulizing gas. Mass spectrometric parameters were optimized for all analytes. Their optimal ionization and fragmentation were listed in Table 3. The main operating parameters of the MS system were given as follows: nebulizer current at 4.0 μA, the symmetrical heaters at 500 °C, both ion source gas (gas 1) and turbo heater gas (gas 2) at 40.0 psig, curtain gas at 20.0 psig and collision gas at medium. Figure 3 displays the optimal precursor ion and product ion of each analyte. In addition, dwell time was set at 100 ms to detect all analytes.

### 4.5. Method Validation

Method validation was performed according to the guidance as specified by the Food and Drug Administration, USA [41]. Method validation was implemented in pure standards solutions spiked into 10 mg blank hair matrix collected from healthy volunteers without taking any drugs containing nevirapine. The analyte-free, interference-free blank matrix was treated in a manner slightly different from that for the actual patients’ hair samples. Before being incubated in methanol, the blank matrix was mixed with 50 μL working solutions of standards, 50 μL IS’s working solutions and 900 μL methanol. Then the blank matrix with 1 mL solution was treated with the same method as patients’ hair samples were. The final standard concentrations of calibrated curves were 4, 10, 20, 100, 200, 400, 800, 2000, 4000, 10,000, 20,000, 40,000 and 50,000 pg/mg for NVP, and 2, 4, 10, 20, 100, 200, 400, 800, 1000 and 1250 pg/mg for both 2-OH NVP and 3-OH NVP.

Limit of detection (*LOD*) was determined at the ratio of signal to noise of 3 (*S*/*N* = 3) and it represented the qualitative detection of the minimum concentration or minimum amount of the substance to be measured from the sample. In a similar way, limit of quantitation (*LOQ*) was determined at *S*/*N* = 10 and it represented the quantitative detection of the minimum concentration or minimum amount of the substance to be measured from the sample. Intra-day precision was evaluated by CV through repeating it 5 times within a day and inter-day precision was evaluated by CV using 5 replicates over 5 consecutive days. Intra-day and inter-day precisions were detected at *LOQ*, low, medium and high concentrations (low was 2–3 times the *LOQ*, medium was at 40–50% the upper limit of quantification (UOQ) and high was at 80% UOQ), namely, 11, 30, 20,000 and 40,000 pg/mg for NVP, and 6, 15, 500 and 1000 pg/mg for both 2-OH NVP and 3-OH NVP. Recovery referred to the recovery of the experimental method, which was reported as a percentage of the concentration of the standard analytes evaluated with the calibration curve versus the actual spiked concentration of standard analyte. Recovery was established with three independent runs at low, medium and high concentrations, namely, 30, 20,000 and 40,000 pg/mg for NVP, and 15, 500 and 1000 pg/mg for both 2-OH NVP and 3-OH NVP.

Matrix effect was assessed by using six lots of blank hair matrices to calculate the matrix factor (*MF*) for external standards (ESs), IS and IS normalized *MF* for ESs. The six blank matrices were spiked with the analytes in triplicate in one batch for NVP, 2-OH NVP and 3-OH NVP. Selectivity was assessed with six lots of blank hair matrices spiked into drugs at the *LOQ* level to confirm whether the endogenous compounds of the blank matrix interfered with determining the analyte or internal standards.

Stability was assessed by the changing situation between the concentration acquired by calibrated curve and the actual concentration of the standards under different conditions in blank hair matrix at low and high concentrations in triplicate. Concretely, benchtop stability referred to the stability of the above two levels solutions (the supernatant obtained after the centrifugation) in blank matrix stored in an ice bucket at 4 °C for 12 h. Stock solution stability meant the stability of keeping the solutions at the temperature of the reserve solution (−20 °C) for 24 h. Autosampler stability referred to the stability of solutions stored at 10 °C simulating auto-sampler condition for 12 h. Freeze-thaw stability required the process of three freeze-thaw cycles, and the solutions (the supernatant obtained after the centrifugation) were frozen for at least 12 h between cycles where frozen solutions were permitted to thaw at ambient temperature for 2 h. Long-term stability was evaluated in a refrigerator at 4 °C for a week.

### 4.6. Statistical Analysis

The statistical packages SPSS 25.0 and Origin 2018 were utilized for comprehensive statistical analysis. Linearly fitting curves were graphed using Origin 2018 analytical software. Seventy-five HIV-positive patients were divided into two groups with over 25 days and less than 26 days of oral drug administration per month (high- and low-adherence groups) as listed in Table 2. Chi-square test and *t*-test for two independent samples were done to compare whether there were significant differences in gender distribution, the concentrations of the three compounds in hair and age between the two groups. Univariate analysis of variance was conducted for confirming the inter-group differences in the three compounds in hair when gender was used as a covariate. Additionally, Mann-Whitney *U*-test for two independent samples was implemented to examine the gender differences in the concentrations of the three compounds in hair, days of NVP oral administration and age for the two groups. Pearson correlation analysis was performed for examining the associations between the concentrations of NVP, 2-OH NVP and 3-OH NVP in hair.

## 5. Conclusions

This study has developed a specific, sensitive and selective method for simultaneous quantification of NVP and its two main metabolites (i.e., 2-OH NVP and 3-OH NVP) in human hair. The method was developed with LC-MS/MS coupled with an APCI in positive mode and MRM. The method displayed good performances in linearity, intra-day and inter-day CVs, recovery, selectivity and stability. More importantly, this study found that NVP’s metabolites showed high consistency with NVP in evaluating long-term adherence although they showed moderate correlations with NVP. This makes it necessary to consider the antiretroviral drug’s metabolites together with the prototype when the concentrations of drugs in hair are used as a critical method for measuring long-term adherence to antiretroviral drugs. At the same time, it also needs to be confirmed through quantifying prototypes and the metabolites of more antiretroviral drugs in future studies.

## Figures and Tables

**Table 1:** Linearity, limits of detection and quantitation (*LOD* and *LOQ*), retention time, intra-day and inter-day coefficients of variation and recovery for NVP, 2-OH NVP and 3-OH NVP in human hair.

	NVP	2-OH NVP	3-OH NVP
Calibration curve	y = 0.00534x + 0.00799	y = 0.00308x − 0.00136	y = 0.00354x − 0.00316
*R* ^2 a^	0.9997	0.9997	0.9987
Linear Range (pg/mg)	11–50,000	6–1250	6–1250
*LOD* (pg/mg) ^b^	3	2	2
*LOQ* (pg/mg) ^b^	11	6	6
Retention time (min)	7.84	6.41	6.92
*CV* (%) of the retention times ^c^	3.8	3.2	4.5
Intra-day *CV* (%, *n* = 5) ^d,e^			
*LOQ*	8.5	5.4	10.2
Low	5.8	2.8	4.3
Medium	3.5	7.6	3.2
High	2.2	4.2	5.6
Inter-day *CV* (%, *n* = 5) ^d,e^			
*LOQ*	7.6	10.5	7.8
Low	8.8	5.2	6.4
Medium	9.4	3.1	3.6
High	2.3	5.6	8.1
Recovery (%, *n* = 5) ^d^			
Low	105.2 ± 4.2	101.3 ± 4.4	100.5 ± 3.1
Medium	106.8 ± 3.5	99.5 ± 6.8	102.2 ± 3.2
High	105.6 ± 3.2	103.5 ± 5.8	103.9 ± 6.0

^a^*R*^2^ represents the square of correlation coefficient. ^b^ Limit of detection (*LOD*) was set at the ratio of signal to noise (*S*/*N*) of 3, limit of quantitation (*LOQ*) was set at *S*/*N* = 10. ^c^ *CV* (%) of the retention times between samples and blank matrix + standard = (Mean_the retention times of sample__s_ − Mean_the retention times of blank matrix + standard_)/Mean_the retention times of blank matrix + standard_ × 100%. ^d^ *LOQ*, low, medium and high concentrations were 11, 30, 20,000 and 40,000 pg/mg for NVP; 6, 15, 500 and 1000 pg/mg for both 2-OH NVP and 3-OH NVP. ^e^ Intra-day and inter-day precisions were estimated by intra-day and inter-day coefficients of variation (CV).

**Table 2:** The comparison between high- and low-adherence groups in gender, age, the days of NVP oral administration and the concentrations of NVP, 2-OH NVP and 3-OH NVP in hair.

	High Adherence Group ^a^	Low Adherence Group ^a^	Statistical Value ^b^
Number	61	14	-
Gender (female/male)	54/7	8/6	*χ^2^* = 7.826 **
Age (years) ^c^	43.39 ± 9.34, 25–67	45.29 ± 6.65, 27–52	*t*_66_ = −0.713
Adherence (days/month) ^d^	29.85 ± 0.65, 27–30	18.43 ± 4.62, 10–25	*t*_13.120_ = 9.232 ***
NVP concentration(pg/mg) ^e,f^	30,600 ± 12,346, 3–53,218	5380 ± 13,694, 3–42,221	*t*_73_ = 6.755 ***
2-OH NVP concentration (pg/mg) ^e,g^	216 ± 212, 2–1055	24 ± 57, 0–166	*t*_71.141_ = 6.137 ***
3-OH NVP concentration (pg/mg) ^e,h^	270 ± 253, 0–1235	35 ± 80, 0–235	*t*_66.15_ = 6.061 ***

Notes: ** *p* < 0.01, *** *p* < 0.001. ^a^ High adherence means days of oral administration every month >25 days, and low adherence means the days ≤25 days. ^b^ Gender distribution was compared with Chi-square and the other variables were compared with *t*-test for two independent samples. ^c^ Seven participants in the high adherence group were excluded because they missed the information on age. ^d^ It refers to how many days HIV-patients used antiretroviral drugs by oral administration over the last month and is presented as *M* ± *SD* and range where *M* was mean and *SD* was standard deviation. ^e^ The concentration was presented as *M* ± *SD* and range where the concentration was below *LOQ*, but over *LOD* was set as *LOD* and the concentration below *LOD* as 0. ^f^ One participant in the high adherence group and two participants in the low adherence group had hair NVP concentrations below *LOQ*, but over *LOD*. ^g^ Two participants in the high adherence group and six participants in the low adherence group had hair 2-OH NVP concentrations below *LOQ*, but over *LOD*. Four participants in the low adherence group had hair 2-OH NVP concentrations below *LOD*. ^h^ One participant in the high adherence group and five participants in the low adherence group had hair 3-OH NVP concentrations below *LOQ*, but over *LOD*. One participant in the high adherence group and four participants in the low adherence group had hair 3-OH NVP concentrations below *LOD*.

**Table 3:** Optimal conditions for ionization and fragmentation of the three compounds and their ISs.

Analyte	Precursor Ion and Product Ion (*m*/*z*)	DP(V)	EP(V)	CEP(V)	CE(V)	CXP(V)
NVP *	267.1/226.3	54.01	5.38	21.30	37.39	3.42
NVP	267.1/198.3	54.01	5.38	21.30	50.75	3.08
NVP-d3	270.1/229.4	63.43	5.93	24.97	36.61	3.48
2-OH NVP *	283.0/161.1	56.21	6.47	43.12	33.26	2.69
2-OH NVP	283.0/238.1	56.21	6.47	43.12	30.03	5.11
2-OH NVP-d3	285.9/161.0	62.92	5.58	22.34	34.12	1.74
3-OH NVP *	283.0/214.3	68.80	5.60	38.13	45.17	3.72
3-OH NVP	283.0/243.3	68.80	5.60	38.13	39.54	4.35

* The most sensitive transition was used for quantitation and the other one was employed for confirmation. DP, declustering potential; EP, entrance potential; CEP, collision cell entrance potential; CE, collision energy; CXP, collision cell exit potential.

## Supplementary Materials

The following are available online, Table S1: Matrix factors (*MF*) and its coefficients of variation (CV_MF_) for NVP, 2-OH NVP and 3-OH NVP in human hair; Table S2: Sample selectivity for NVP, 2-OH NVP, 3-OH NVP and their IS in human hair; Table S3: The results on benchtop, stock solution, autosampler, freeze thaw, long term stability at low and high concentrations for NVP, 2-OH NVP and 3-OH NVP.

## References

[1] De Bethune M.-P. (2010). Non-nucleoside reverse transcriptase inhibitors (NNRTIs), their discovery, development, and use in the treatment of HIV-1 infection: A review of the last 20 years (1989–2009). Antivir. Res..

[2] De Clercq E. (2004). Non-nucleoside reverse transcriptase inhibitors (NNRTIs): Past, present, and future. Chem. Biodivers..

[3] De Clercq E. (2009). Anti-HIV drugs: 25 compounds approved within 25 years after the discovery of HIV. Int. J. Antimicrob. Agents.

[4] Kappelhoff B.S., van Leth F., MacGregor T.R., Lange J.M.A., Beijnen J.H., Huitema A.D.R., Grp N.N.S. (2005). Nevirapine and efavirenz pharmacokinetics and covariate analysis in the 2NN study. Antivir. Ther..

[5] Palella F.J., Delaney K.M., Moorman A.C., Loveless M.O., Fuhrer J., Satten G.A., Aschman D.J., Holmberg S.D., Investigators H.I.V.O.S. (1998). Declining morbidity and mortality among patients with advanced human immunodeficiency virus infection. N. Engl. J. Med..

[6] Pereira S.A., Wanke R., Marques M.M., Monteiro E.C., Antunes A.M.M., Fishbein J.C. (2012). Insights into the Role of Bioactivation Mechanisms in the Toxic Events Elicited by Non-nucleoside Reverse Transcriptase Inhibitors. Advances in Molecular Toxicology.

[7] Pollard R.B., Robinson P., Dransfield K. (1998). Safety profile of nevirapine, a nonnucleoside reverse transcriptase inhibitor for the treatment of human immunodeficiency virus infection. Clin. Ther..

[8] Robbins R.N., Spector A.Y., Mellins C.A., Remien R.H. (2014). Optimizing ART adherence: Update for HIV treatment and prevention. Curr. HIV/AIDS Rep..

[9] Pasternak A.O., de Bruin M., Jurriaans S., Bakker M., Berkhout B., Prins J.M., Lukashov V.V. (2012). Modest Nonadherence to Antiretroviral Therapy Promotes Residual HIV-1 Replication in the Absence of Virological Rebound in Plasma. J. Infect. Dis..

[10] Yan J., Liu J., Su B., Pan X.H., Wang Z., Wu J.J., Zhang J.F., Ruan Y.H., Hsi J., Liao L.J. (2016). Lamivudine Concentration in Hair and Prediction of Virologic Failure and Drug Resistance among HIV Patients Receiving Free ART in China. PLoS ONE.

[11] Zhang Q., Qiao S., Yang X., Li X. (2019). Antiretroviral Concentration in Hair as a Measure for Antiretroviral Medication Adherence: A Systematic Review of Global Literature. AIDS Behav..

[12] Avataneo V., D’Avolio A., Cusato J., Cantu M., De Nicolo A. (2019). LC-MS application for therapeutic drug monitoring in alternative matrices. J. Pharm. Biomed. Anal..

[13] Gandhi M., Greenblatt R.M. (2002). Hair it is: The long and short of monitoring antiretroviral treatment. Ann. Intern. Med..

[14] Pragst F., Balikova M.A. (2006). State of the art in hair analysis for detection of drug and alcohol abuse. Clin. Chim. Acta.

[15] Gandhi M., Bacchetti P., Ofokotun I., Jin C., Ribaudo H.J., Haas D.W., Sheth A.N., Horng H., Phung N., Kuncze K. (2019). Antiretroviral Concentrations in Hair Strongly Predict Virologic Response in a Large Human Immunodeficiency Virus Treatment-naive Clinical Trial. Clin. Infect. Dis..

[16] Gandhi M., Glidden D.V., Liu A., Anderson P.L., Horng H., Defechereux P., Guanira J.V., Grinsztejn B., Chariyalertsak S., Bekker L.G. (2015). Strong correlation between concentrations of tenofovir (TFV) emtricitabine (FTC) in hair and TFV diphosphate and FTC triphosphate in dried blood spots in the iPrEx open label extension: Implications for pre-exposure prophylaxis adherence monitoring. J. Infect. Dis..

[17] Gandhi M., Glidden D.V., Mayer K., Schechter M., Buchbinder S., Grinsztejn B., Hosek S., Casapia M., Guanira J., Bekker L.G. (2016). Association of age, baseline kidney function, and medication exposure with declines in creatinine clearance on pre-exposure prophylaxis: An observational cohort study. Lancet HIV.

[18] Gandhi M., Murnane P.M., Bacchetti P., Elion R., Kolber M.A., Cohen S.E., Horng H., Louie A., Kuncze K., Koss C.A. (2017). Hair levels of preexposure prophylaxis drugs measure adherence and are associated with renal decline among men/transwomen. AIDS.

[19] Koss C.A., Hosek S.G., Bacchetti P., Anderson P.L., Liu A.Y., Horng H., Benet L.Z., Kuncze K., Louie A., Saberi P. (2018). Comparison of Measures of Adherence to Human Immunodeficiency Virus Preexposure Prophylaxis among Adolescent and Young Men Who Have Sex with Men in the United States. Clin. Infect. Dis..

[20] Koss C.A., Natureeba P., Mwesigwa J., Cohan D., Nzarubara B., Bacchetti P., Horng H., Clark T.D., Plenty A., Ruel T.D. (2015). Hair concentrations of antiretrovirals predict viral suppression in HIV-infected pregnant and breastfeeding Ugandan women. AIDS.

[21] Liu A.Y., Yang Q., Huang Y., Bacchetti P., Anderson P.L., Jin C., Goggin K., Stojanovski K., Grant R., Buchbinder S.P. (2014). Strong relationship between oral dose and tenofovir hair levels in a randomized trial: Hair as a potential adherence measure for pre-exposure prophylaxis (PrEP). PLoS ONE.

[22] Pintye J., Bacchetti P., Teeraananchai S., Kerr S., Prasitsuebsai W., Singtoroj T., Kuncze K., Louie A., Koss C.A., Jin C. (2017). Lopinavir hair concentrations are the strongest predictor of viremia in HIV-infected asian children and adolescents on second-line antiretroviral therapy. J. Acquir. Immune Defic. Syndr..

[23] Abaasa A., Hendrix C., Gandhi M., Anderson P., Kamali A., Kibengo F., Sanders E.J., Mutua G., Bumpus N.N., Priddy F. (2018). Utility of Different Adherence Measures for PrEP: Patterns and Incremental Value. AIDS Behav..

[24] Baxi S.M., Vittinghoff E., Bacchetti P., Huang Y., Chillag K., Wiegand R., Anderson P.L., Grant R., Greenblatt R.M., Buchbinder S. (2018). Comparing pharmacologic measures of tenofovir exposure in a U.S. pre-exposure prophylaxis randomized trial. PLoS ONE.

[25] Seifert S.M., Castillo-Mancilla J.R., Erlandson K., Morrow M., Gandhi M., Kuncze K., Horng H., Zheng J.H., Bushman L.R., Kiser J.J. (2018). Brief Report: Adherence Biomarker Measurements in Older and Younger HIV-Infected Adults Receiving Tenofovir-Based Therapy. J. Acquir. Immune Defic. Syndr..

[26] Erickson D.A., Mather G., Trager W.F., Levy R.H., Keirns J.J. (1999). Characterization of the in vitro biotransformation of the HIV-1 reverse transcriptase inhibitor nevirapine by human hepatic cytochromes P-450. Drug Metab. Dispos..

[27] Marinho A.T., Miranda J.P., Caixas U., Charneira C., Goncalves-Dias C., Marques M.M., Monteiro E.C., Antunes A.M.M., Pereira S.A. (2019). Singularities of nevirapine metabolism: From sex-dependent differences to idiosyncratic toxicity. Drug Metab. Rev..

[28] Rowland L.S., MacGregor T.R., Campbell S.J., Jenkins R., Pearsall A.B., Morris J.P. (2007). Quantitation of five nevirapine oxidative metabolites in human plasma using liquid chromatography-tandem mass spectrometry. J. Chromatogr. B Analyt. Technol. Biomed. Life Sci..

[29] Riska P., Lamson M., MacGregor T., Sabo J., Hattox S., Pav J., Keirns J. (1999). Disposition and biotransformation of the antiretroviral drug nevirapine in humans. Drug Metab. Dispos..

[30] Wu Y., Yang J., Duan C., Chu L., Chen S., Qiao S., Li X., Deng H. (2018). Simultaneous determination of antiretroviral drugs in human hair with liquid chromatography-electrospray ionization-tandem mass spectrometry. J. Chromatogr. B Analyt. Technol. Biomed. Life Sci..

[31] Chu L., Wu Y., Duan C., Yang J., Yang H., Xie Y., Zhang Q., Qiao S., Li X., Shen Z. (2018). Simultaneous quantitation of zidovudine, efavirenz, lopinavir and ritonavir in human hair by liquid chromatography-atmospheric pressure chemical ionization-tandem mass spectrometry. J. Chromatogr. B Analyt. Technol. Biomed. Life Sci..

[32] Olds P.K., Kiwanuka J.P., Nansera D., Huang Y., Bacchetti P., Jin C., Gandhi M., Haberer J.E. (2015). Assessment of HIV antiretroviral therapy adherence by measuring drug concentrations in hair among children in rural Uganda. AIDS Care Psychol. Socio-Med. Asp. AIDS/HIV.

[33] Huang Y., Yang Q., Yoon K., Lei Y., Shi R., Gee W., Lin E.T., Greenblatt R.M., Gandhi M. (2011). Microanalysis of the antiretroviral nevirapine in human hair from HIV-infected patients by liquid chromatography-tandem mass spectrometry. Anal. Bioanal. Chem..

[34] Ren C., Fan-Havard P., Schlabritz-Loutsevitch N., Ling Y., Chan K.K., Liu Z. (2010). A sensitive and specific liquid chromatography/tandem mass spectrometry method for quantification of nevirapine and its five metabolites and their pharmacokinetics in baboons. Biomed. Chromatogr..

[35] Liu Z., Fan-Havard P., Xie Z., Ren C., Chan K.K. (2007). A liquid chromatography/atmospheric pressure ionization tandem mass spectrometry quantitation method for nevirapine and its two oxidative metabolites, 2-hydroxynevirapine and nevirapine 4-carboxylic acid, and pharmacokinetics in baboons. Rapid Commun. Mass Spectrom..

[36] Smeraglia J., Baldrey S.F., Watson D. (2002). Matrix effects and selectivity issues in LC-MS-MS. Chromatographia.

[37] Dams R., Huestis M.A., Lambert W.E., Murphy C.M. (2003). Matrix effect in bio-analysis of illicit drugs with LC-MS/MS: Influence of ionization type, sample preparation, and biofluid. J. Am. Soc. Mass Spectrom..

[38] Niessen W.M., Manini P., Andreoli R. (2006). Matrix effects in quantitative pesticide analysis using liquid chromatography-mass spectrometry. Mass Spectrom. Rev..

[39] Matuszewski B.K. (2006). Standard line slopes as a measure of a relative matrix effect in quantitative HPLC-MS bioanalysis. J. Chromatogr. B Analyt. Technol. Biomed. Life Sci..

[40] Souverain S., Rudaz S., Veuthey J.-L. (2004). Matrix effect in LC-ESI-MS and LC-APCI-MS with off-line and on-line extraction procedures. J. Chromatogr. A.

[41] Food and Drug Administration USA Bioanalytical Method Validation Guidance for Industry. https://www.fda.gov/regulatory-information/search-fda-guidance-documents/bioanalytical-method-validation-guidance-industry.

