# Supplementary material for: LC-MS/MS Quantification of Nevirapine and Its Metabolites in Hair for Assessing Long-Term Adherence
Source: Molecules. 2020 Dec 2;25(23):5692. doi: 10.3390/molecules25235692 (PMC7730356; doi:10.3390/molecules25235692)
Supplement: Supplementary file 1 [file molecules-25-05692-s001.pdf]

Supplement materials

Table S1 Matrix factors (*MF*) and its coefficients of variation (CV_{MF}) for NVP, 2-OH NVP and 3-OH NVP in human hair.

	Lot A	Lot B	Lot C	Lot D	Lot E	Lot F	Mean	SD	CV_{MF} (%)
MF for ESs [a]									
NVP	0.90	1.05	1.01	0.85	1.09	0.97	0.98	0.09	9.2
2-OH NVP	1.06	1.09	0.86	1.10	1.01	0.94	1.01	0.10	9.5
3-OH NVP	0.94	1.14	0.79	1.16	0.95	0.94	0.99	0.14	14.1
MF for IS [b]									
NVP-d3	0.85	0.98	1.03	0.76	0.97	1.00	0.93	0.10	11.0
2-OH NVP-d3	0.97	1.21	0.89	1.09	0.93	1.00	1.01	0.12	11.5
IS normalized *MF* for ESs [c]									
NVP	1.06	1.09	0.99	1.12	1.14	0.97	1.06	0.07	6.5
2-OH NVP	1.10	0.91	0.97	1.01	1.08	0.98	1.01	0.07	7.2
3-OH NVP	0.97	0.94	0.89	1.07	1.02	0.99	0.98	0.06	6.1

[a] *MF* for ESs was calculated as the ratio of the ES's peak area in blank hair matrix to the ES's peak area in pure solution without blank hair matrix.

[b] *MF* for IS was defined as the ratio of the IS's peak area in blank hair matrix to the IS's peak area in pure solution without blank hair matrix.

[c] IS normalized *MF* was defined as the ratio of ES's *MF* to IS's *MF*.

Table S2 Sample selectivity for NVP, 2-OH NVP, 3-OH NVP and their IS in human hair.

	Lot A	Lot B	Lot C	Lot D	Lot E	Lot F	Mean	SD
Selectivity for ESs [a] (%)								
NVP	7.9	4.6	3.9	3.9	5.0	4.1	4.9	1.5
2-OH NVP	7.4	6.3	4.4	12.1	6.6	5.5	7.0	2.7
3-OH NVP	6.6	4.5	3.4	8.8	6.7	4.1	5.7	2.0
Selectivity for IS [a] (%)								
NVP-d3	0.1	0.4	0.5	1.4	0.7	0.8	0.6	0.5
2-OH NVP-d3	0.7	0.7	1.8	1.5	0.7	0.7	1.0	0.5

[a] Selectivity for ESs or IS was defined as the ratio of peak areas of co-eluting compounds from blank hair matrices to the peak area of the analytes at the *LOQ* level.

Table S3The results on benchtop, stock solution, autosampler, freeze-thaw, long-term stability at low and high concentrations for NVP, 2-OH NVP and 3-OH NVP.

Analyte	Nominal (pg/mg)	Mean (pg/mg)	*DEV* (%) [a]	*CV* (%) [b]
Benchtop stability				
NVP	30	28	-6.2	2.8
	40000	40556	1.4	1.7
2-OH NVP	15	16	4.5	4.5
	1000	1080	8.0	2.0
3-OH NVP	15	14	-9.8	6.2
	1000	1040	4.0	3.2
Stock solution stability				
NVP	30	32	8.4	4.3
	40000	45093	12.7	2.2
2-OH NVP	15	14	-4.3	8.2
	1000	1124	12.4	5.2
3-OH NVP	15	16	4.8	12.1
	1000	1116	11.6	5.2
Autosampler stability				
NVP	30	33	8.7	3.0
	40000	43972	9.9	1.6
2-OH NVP	15	16	5.4	1.7
	1000	1125	12.5	2.5
3-OH NVP	15	16	8.3	1.9
	1000	1123	12.3	8.2
Freeze-thaw stability				
NVP	30	33	10.6	1.8
	40000	44092	10.2	1.6
2-OH NVP	15	16	6.3	1.9
	1000	1105	10.5	1.4
3-OH NVP	15	14	-4.5	5.4
	1000	1084	8.4	1.6
Long-term stability				
NVP	30	32	6.5	1.2
	40000	38569	-3.6	5.6
2-OH NVP	15	16	4.3	5.2
	1000	1081	8.08	2.3
3-OH NVP	15	14	-8.7	3.3
	1000	1034	3.4	1.7

[a] deviation (*DEV*) = $\frac{\bar{x}-x_o}{x_o} * 100\%$ where $\bar{x}$ represents the average value of the tested group and x_o represents the true value of the measured data.

[b] coefficient of variation (*CV*) = $\frac{S}{\bar{x}} * 100\%$ where $\bar{x}$ represents the average value of the tested group and *S* represents the standard deviation of the measured data.
